# Chinese provincial multi-regional input-output database for 2012, 2015, and 2017

**DOI:** 10.1038/s41597-021-01023-5

**Published:** 2021-09-22

**Authors:** Heran Zheng, Yangchun Bai, Wendong Wei, Jing Meng, Zhengkai Zhang, Malin Song, Dabo Guan

**Affiliations:** 1 Industrial Ecology Programme, Department of Energy and Process Engineering, Norwegian University of Science and Technology, Trondheim, 7491 Norway; 2 Institute of Blue and Green Development, Shandong University, Weihai, 264209 China; 3 School of International and Public Affairs, Shanghai Jiao Tong University, Shanghai, 200030 China; 4 The Bartlett School of Sustainable Construction, University College London, London, WC1H 0QB UK; 5 College of Management and Economics, Tianjin University, Tianjin, 300072 China; 6 School of Statistics and Applied Mathematics, Anhui University of Finance and Economics, Bengbu, 233030 People’s Republic of China; 7 Department of Earth System Science, Ministry of Education Key Laboratory for Earth System Modelling, Tsinghua University, 100084 Beijing, China

**Keywords:** Economics, Environmental economics

## Abstract

Global production fragmentation generates indirect socioeconomic and environmental impacts throughout its expanded supply chains. The multi-regional input-output model (MRIO) is a tool commonly used to trace the supply chain and understand spillover effects across regions, but often cannot be applied due to data unavailability, especially at the sub-national level. Here, we present MRIO tables for 2012, 2015, and 2017 for 31 provinces of mainland China in 42 economic sectors. We employ hybrid methods to construct the MRIO tables according to the available data for each year. The dataset is the consistent China MRIO table collection to reveal the evolution of regional supply chains in China’s recent economic transition. The dataset illustrates the consistent evolution of China’s regional supply chain and its economic structure before the 2018 US-Sino trade war. The dataset can be further applied as a benchmark in a wide range of in-depth studies of production and consumption structures across industries and regions.

## Abstract (summary)

**Table:** 

Measurement(s)	multi-regional input-output
Technology Type(s)	partial survey
Factor Type(s)	province • year
Sample Characteristic - Location	China

Machine-accessible metadata file describing the reported data: 10.6084/m9.figshare.15362565

## Background & Summary

Following the 2008 financial recession, China entered an economic transition period (“New Normal”), during which growth patterns shifted from the old investment-driven pattern into a new growth paradigm characterised by “high quality but lower growth”. The paradigm aimed to lift China’s position of global value chains by prioritising the development of high value-added manufacturing and services^1–5^. The changes that came about in the economic transition were huge, not only in the national economic structure but more in the inter-provincial supply chains. Given China’s vast territory and the variations in socioeconomic development, tracing the evolution of cross-province supply chains is of great importance for regional development policy-making as well as for understanding the environmental spill-over effects along the supply chains^6,7^. From a global perspective, China’s economic transition from 2012–2017 is historically significant in that it occurred between the post-financial crisis and the 2018 US-Sino trade war. The period provides a benchmark for further studies about how the US-Sino trade war reshaped China’s economic structure and regional supply chains.

The multi-region input-output model (MRIO) is the dominant model used to quantify spillover effects through supply chains and regional heterogeneity^8–11^. Many scholars have constructed MRIO tables in the past decade, especially at the international level. The most commonly used MRIO tables in the international scientific community are GTAP^12,13^, WIOD^8^, EORA^14^, OECD-ICIO^15^ and EXIOBASE^16^. In China, several provincial MRIO tables are already available (Table 1). Li *et al*. in DRC (Development Research Centre) constructed the consistent provincial MRIO tables every five years from 1997 to 2012^17,18^; Zhang (2012) of SIC (State Information Centre) built MRIO tables for eight regions based on the entropy model for 1997, 2002 and 2007^19^; Liu of IGSNRR (Institute of Geographic Sciences and Natural Resources Research-China Academy of Sciences) constructed provincial MRIO tables for 2007, 2010, and 2012 adjusted by geographic indicators^20–23^; Mi of CEADs (China Carbon Emissions Databases) group adopt the same method to construct an MRIO table for 30 provinces with 30 sectors^24^; Zhang and colleagues of RCFEDS (Research Centre on Fictitious Economy and Data Science) compiled an MRIO table for 30 provinces with the highest sector resolution of 60 sectors, but only for 2002^25^. Despite these collective efforts, all datasets are compiled using different methods, assumptions, resolution or coverage of sectors, regions and time coverage, resulting in a lack of coherent and consistent data and making it difficult to cross-reference^26^. For example, IGSNRR adjusts the gravity model using parameters such as spatial weights and competitive coefficients. The spatial weights are to take into account the effects of trade between neighbouring places on focal trade, while competitive coefficients are to reduce the trade of the same product between two places; DRC adjusts the trade data of the original SRIO table by using Chinese customs datasets. Such inconsistencies mean that it is very difficult to make time-series or trend analyses. Most importantly, all available datasets are constructed at most to 2012 and so cannot trace the evolution of interregional supply chains during the economic transition. In the post-2012 period, to our knowledge, only our previous work which reveals the carbon flows in 2015 has illustrated the interregional supply chains for 2015^4^, while the MRIO table for 2017 has yet to be constructed. The lack of an updated dataset that reflects the evolutionary supply chain deeply undermines our understanding of the heterogeneous effects of China’s economic transition at the regional level. It is worth noting that the tables compiled by this study are just to bridge the gap in terms of the lack of recent MRIO tables, and our study also contributes to understanding China’s regional heterogeneity alone with other institutes.

**Table 1:** The list of Chinese MRIO database.

Institutes/Group	Regions	Sectors	Year
Development Research Centre of State Council (DRC)	30 Provinces 31 Provinces (2012)	33 Sectors 42 sectors (2012)	1997,2002,2007,2012
State Information Centre (SIC)	8 regions	30 Sectors (1997) 17 Sectors (2002, 2007)	1997,2002,2007
Institute of Geographic Science and Natural Resources Research, CAS (IGSNRR)	30 provinces 31 provinces (2012)	33 Sectors 42 sectors (2012)	2007,2010,2012
Research Centre on Fictitious Economy and Data Science, CAS (RCFEDS)	30 provinces	60 Sectors	2002
China Carbon Emissions Databases (CEADs)	30 provinces	30 Sectors	2012

To bridge the data gap, we present a collection of provincial MRIO tables for 2012, 2015, and 2017 that cover China’s economic transition period constructed using a consistent approach. We compiled MRIO tables for the three years and include 42 sectors of all 31 provinces of mainland China, excluding Hongkong, Macao and Taiwan, by the maximum entropy model. The MRIO table construction was based on official data, including provincial single region IO tables (SRIO), economic data from provincial statistics yearbooks and China’s customs database. The unified formats of the MRIO tables are compatible with China’s national SRIO table, with identical sector classification and five categories in the final demands: rural household consumption, urban household consumption, government consumption, capital formation, and inventory changes. This dataset can be widely used in both economic analyses to identify the driving forces of regional growth in China’s economic transition and environmental impacts focusing on the interregional spill-over effects along the supply chains.

## Methods

China’s provincial MRIO tables for 2012, 2015, and 2017 were compiled using a partial survey approach, which combines the official survey data and modelled outcomes^6,27^. The partial survey approach allows MRIO table construction to be regarded as linking provincial SRIO tables with the trade matrix for each sector. Provincial SRIO tables are often available from surveyed data, while the trade matrix for sectors are unavailable and rely on modelling. However, there are two challenges to be faced before compilation. On the one hand, due to the high costs for the ad hoc survey for input-output table construction, China’s official provincial SRIO tables are only released every five years, with the year ending with 2 or 7 (e.g. 2012 and 2017 in this case). The SRIO table for years ending with 5 (e.g. 2015) is not compulsory. Hence, SRIO tables of 2012 and 2017 were available for all provinces whereas SRIO tables for 2015 were not. Thus, before SRIO tables could be linked to the trade matrix, provincial SRIO tables for 2015 had to be built. In addition, provincial SRIO tables released by the National Statistics Bureau cannot be directly used due to the inconsistent trade flows between provinces, which is the case for 2012 and 2017. For a given product, the total domestic exports should be equal to the total domestic imports in an economy but officially released SRIO tables often fail to meet this condition. In this study, a cross-entropy model is thus employed to address these problems. The model follows the minimal cross-entropy principle (or Kullback-Leibler divergence) to minimise the entropy distance between the target and prior distribution^28,29^. The outcome of the cross-entropy model ensures maximum similarity between the target and the known distribution.

**Fig. 1:**
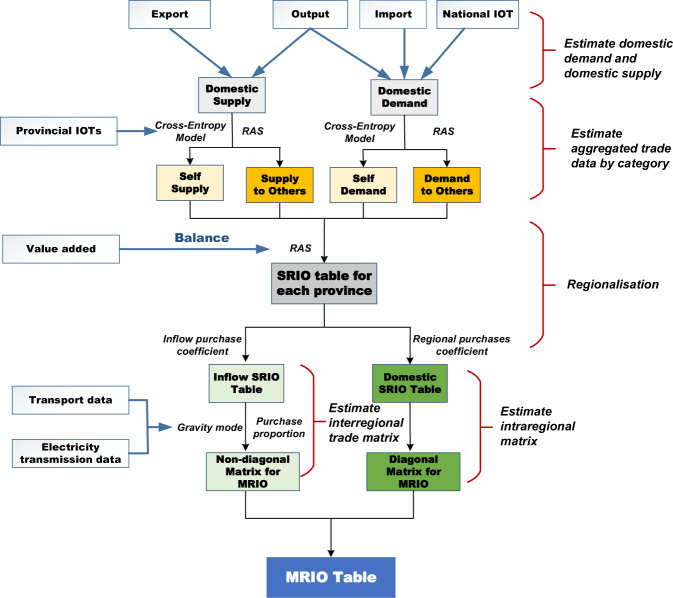
Flowchart of MRIO table construction.

Figure 1 illustrates the 5 steps involved in constructing provincial MRIO tables: (1) Estimation of domestic demand and supply; (2) Disaggregating demand and supply; (3) Adjustment of the provincial SRIO table; (4). Estimation of the interregional trade matrix; (5) Linking adjusted provincial SRIO tables with the trade matrix. Table 2 lists the raw data required in the MRIO table construction. Due to differences in data availability, we introduce two cases according to data treatment processes. Case 1 is based on comprehensive provincial SRIO tables for all 31 provinces, such as 2012 and 2017, while Case 2 is based on incomplete provincial SRIO tables for all provinces, namely, there are no tables for 2015. In short, all of China’s available provincial SRIO tables can be accessed on the websites of 31 provincial statistics bureaus, including all 31 SRIO tables for 2012 and 2017, and a few SRIO tables for 2015. In compiling the model, output and value-added data by sectors can be derived from provincial SRIO tables (in 2012 or 2017 case) or provincial statistical yearbooks (in 2015 case), but provincial statistical yearbooks might not provide the output for tertiary sectors and value-added data for industrial sectors. In this case, we can estimate the missing data based on the assumption of the same share structure of value-added and output. For example, we can estimate value-added data for industrial sectors by multiplying the distribution of their output with the total provincial value-added for industrial sectors. To be consistent with the national SRIO table, aggregated provincial output and value-added by sector for all 31 provinces are scaled by the national value from the national SRIO table. In short, output for tertiary sectors is not available in the yearbooks, but value-added for tertiary sectors is. So, we use the structure of value-added for all provinces to disaggregate the national output of the tertiary sectors (derived from national IOT). Similarly, value-added for industrial sectors is not available in the yearbooks, but the output is. Similarly, we use the output structure for all provinces to disaggregate the national value-added by industrial sectors (derived from national IOT).

**Table 2:** The list of data used in the construction.

Data	Involvement process	Source
Output for sector	Supply estimates	Provincial Statistical Yearbooks
Value-added	GRAS for balancing SRIO	Provincial Statistical Yearbooks
Export and Import	Supply and Demand estimates	China Customs data
Transport data	Gravity Model	National Railway Statistical Yearbook
Electricity transmission data	Gravity Model	China Electric Power Yearbook
Provincial SRIO table for 2012 and 2017	Basic data for 2015 SRIO table	China Statistics Bureau
National SRIO table for 2012 and 2017	Constraint in estimates	China Statistics Bureau

Note: China Customs data are not free for the public, but can be accessed by subscription. National Railway Statistical Yearbook and China Electric Power Yearbook are of hard-copy data and can be accessed by purchasing statistics books published by China Railway and China Electric Power Press. Excepts mentioned data, all other data can be accessed from the website of the local or national Statistics Bureau.

Provincial trade flows (domestic imports and domestic exports) are derived from the China customs database for 2015 or from the official provincial SRIO tables for 2012 and 2017. To estimate the trade matrix, the observed transport data and electricity transmission data were also obtained from national railway statistics and China’s electricity yearbook respectively.

### Estimation of domestic demand and supply

**Fig. 2:**
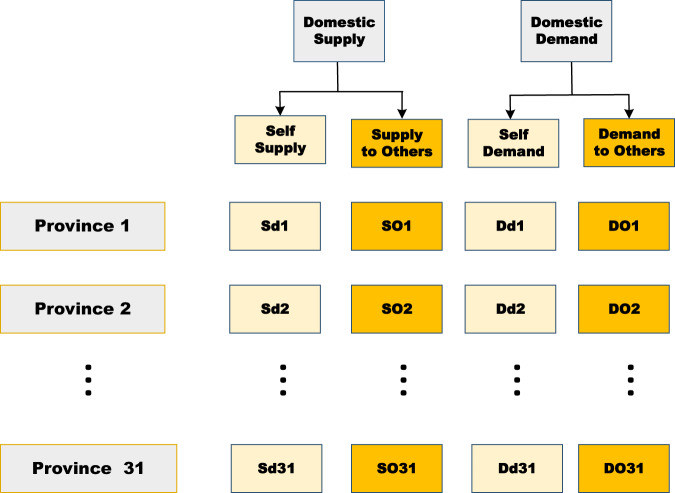
Schematic for supply-demand flow for the province.

The compilation starts with the estimation of supplies and demands (Fig. 2). From the supply perspective, the supply from the given province can be defined by destination and further divided into self-supply, supply to other provinces and supply to other countries (or export). We can estimate domestic supply ${s}_{r}^{i}$ (sector *i* in province *r* supplied to all provinces including itself) by using its total output (${x}_{r}^{i}$) minus exports ($e{x}_{r}^{i}$), as shown in Eq. 1:

1 $$\begin{array}{c}{s}_{r}^{i}={x}_{r}^{i}-e{x}_{r}^{i}\end{array}$$

Where ${x}_{r}^{i}$ refers to the output of commodity *i* in province *r*; $e{x}_{r}^{i}$ refers to the export of commodity *i* in province *r*. ${s}_{r}^{i}$ represents the domestic supply of commodity *i* for province *r*. The demand of a specific province can be defined by the source and further divided into self-demand, demand from other provinces, and demand from other countries (or import). Similarly, we can estimate the domestic demand ${d}_{r}^{i}$ within a province by using its total demand minus imports, where the total demand is the function of intermediate demand (${z}_{r}^{i}$) and final demand (${f}_{r}^{i}$). In case 1, the total demand and imports are available from provincial SRIO tables, and thus shown as Eq. 2. In case 2, the total demands are not available due to the lack of provincial SRIO tables for 2015. We estimated the total demand based on the assumptions: 1. identical technical coefficients between 2012 and 2015 (using the 2012 SRIO table as proxy) to estimate intermediate demand (${z}_{r}^{i\ast }$); 2. The identical proportion of intermediate demands in total demands between 2012 and 2015 (${p}_{r}^{i\ast }$). Where provincial SRIO tables were unavailable for 2015, we used the SRIO tables instead of the 2012 proxy. For Gansu, Anhui, Guangdong, Hunan and Chongqing, we used their provincial SRIO table for 2015. See below for case 1 Eq. (2) and case 2 Eq. (3):

If case 1:

2 $${d}_{r}^{i}={z}_{r}^{i}+{f}_{r}^{i}-i{m}_{r}^{i}$$

If case 2:

3 $$\left\{\begin{array}{c}{z}_{r}^{i\ast }={\sum }_{i}{a}_{201{2}_{r}^{i}}\times {x}_{201{5}_{r}^{i}}\\ {p}_{r}^{i}={\sum }_{i}{z}_{201{2}_{r}^{i}}/t{d}_{201{2}_{r}^{i}}\\ \,t{d}_{r}^{i\ast }={z}_{r}^{i\ast }/{p}_{r}^{i}\\ \widehat{t{d}_{r}^{i}}=\frac{t{d}_{r}^{i\ast }}{{\sum }_{r}t{d}_{r}^{i\ast }}\times n{d}_{201{5}^{i}}\\ {d}_{r}^{i}=\widehat{t{d}_{r}^{i}}-i{m}_{r}^{i}\end{array}\right.$$

Where the domestic demand ${d}_{r}^{i}$ refers to sector *i* needed in province *r*; ${a}_{201{2}_{r}^{i}}$ is the technical coefficients for 2012 for sector *i* needed in province *r*; ${x}_{201{5}_{r}^{i}}$ is the output of sector *i* in province *r*. ${z}_{201{2}_{r}^{i}}$ and $t{d}_{201{2}_{r}^{i}}$ are the intermediate demand and total demand for sector *i* of province *r* for 2012. ${z}_{r}^{i\ast }$ and $t{d}_{r}^{i\ast }$ are preliminary intermediate demand and total demand, where the star * indicates the preliminary. $n{d}_{201{5}^{i}}$ is the national demand for sector *i* in 2015; $i{m}_{r}^{i}$ is the import for sector *i* of province *r*. It is worth noting that the technical coefficients for 2012 were used because the sector classification in the 2012 tables is the same as used in the 2015 tables, while a different classification is used in the 2017 tables (discussed in Table S1). However, choosing different technical coefficients can generate different estimated total demands which lead to different MRIO tables. Therefore, more investigation is needed to address how total demand for each province can be estimated.

### Disaggregating demand and supply

**Fig. 3:**
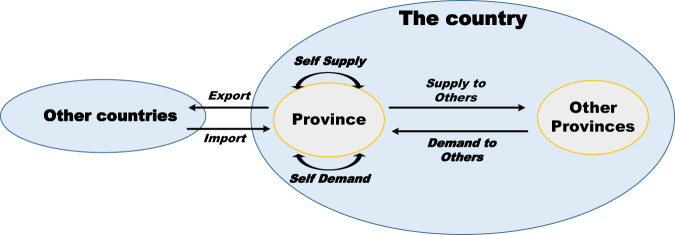
The matrix of supply and demand. For each province, the domestic supply(S) consists of self-supply (SD) and supply to other provinces (SO), whilst domestic demand (D) includes locally supplied demand (DD), and demand from other provinces (DO).

Once domestic supply and demand are established through the above step, we disaggregate the domestic supply and demands by the cross-entropy model (CE), shown in Fig. 3. The cross-entropy model (CE), as mentioned above, is used to obtain the distribution which is closest to the prior information as well as taking into account the given constraints. For a given product or sector, several numeric equations reflect the supply-demand balance, which are constraints in the CE model: (1) the self-supply should be equal to the self-demand for the same provinces; (2) the row sum of Sd and SO should be identical with the domestic supply S. Correspondingly, the row sum of DD and DO should conform with the domestic demand D; (3) the column sum of SO for all provinces should be equal to the column sum of DO for all provinces, as all products giving out are equal to all products received within a certain boundary. Mathematically, this can be shown as:

4 $${\rm{\min }}\;{\rm{C}}\left({\rm{P| | Q}}\right)={\sum }_{i}\,{\sum }_{r}\,{{\rm{p}}}_{ir}\cdot ln\left(\frac{{{\rm{p}}}_{ir}}{{{\rm{q}}}_{ir}}\right)$$

Subject to:

$${\sum }_{i}{\sum }_{j}\left({{\rm{p}}}_{ir}^{{\rm{SD}}}+{{\rm{p}}}_{ir}^{{\rm{SO}}}\right)=1;$$

(the distribution of all supply is equal to 1)

$$\sum _{i}\sum _{r}({{\rm{p}}}_{ir}^{{\rm{DD}}}+{{\rm{p}}}_{ir}^{{\rm{DO}}})=1;$$

(the distribution of all demand is equal to 1)

$$\sum _{r}\,{p}_{i}^{{\rm{SO}}}\times {s}_{i}=\sum _{r}{p}_{i}^{{\rm{DO}}}\times {d}_{i};$$

(the column sum of SO is equal to the sum of DO)

$$\left({p}_{ir}^{{\rm{SD}}}+{p}_{ir}^{{\rm{SO}}}\right)\times {s}_{i}={s}_{ir};$$

(the row sum of domestic supply is equal to the domestic supply by province)

$$\left({p}_{ir}^{{\rm{DD}}}+{p}_{ir}^{{\rm{DO}}}\right)\times {d}_{i}={d}_{ir};$$

(the column sum of domestic demand is equal to the domestic demand by province)

Where ${p}_{ir}$ is the distribution of supply and demand for sector *i* in province *r*; ${q}_{ir}$ is the prior distribution of supply and demand for sector *i* in province *r*. ${s}_{i}$ and ${d}_{i}$ are aggregated domestic supply and demand for sector *i*. ${s}_{ir}$ and ${d}_{ir}$ indicates the domestic supply and demand for sector *i* in province *r*.

### Adjusting provincial single regional input-output table

The above steps re-adjust the domestic supply and demand to make sure that total domestic exports $\left({\sum }_{r}\,s{o}_{r}\right)$ are equal to domestic imports $\left({\sum }_{r}\,s{d}_{r}\right)$ for any product. Thus, we updated the intermediate demand (**Z**) and final demand (**F**) from previous provincial SRIO tables, calibrated with adjusted domestic export and import. We employed the generalised RAS (GRAS) model, which is a variant that allows for non-positive elements in the iterative matrix balancing^30^. For a given SRIO table for province r, two conditions need to be met in terms of the SRIO table balancing. By row, the row sum of intermediate and final demand should be equal to total output minus net export. By column, the column sum of intermediate demand should be equal to total output minus value-added. Mathematically:

5 $$\min {\rm{C}}\left({\rm{P| | Q}}\right)=\sum _{{\rm{i}}}\sum _{{\rm{r}}}\left|{q}_{r}\right|\cdot {p}_{r}\cdot ln\left(\frac{{p}_{r}}{e}\right)$$

Subject to:

$${\sum }_{{\rm{j}}}+{q}_{r}^{ij}\cdot {p}_{r}^{ij}={X}_{r}^{j}-V{A}_{r}^{j}$$

(column constraint)

$${\sum }_{{\rm{i}}}\,{q}_{r}^{ij}\cdot {p}_{r}^{ij}={X}_{r}^{i}-N{E}_{r}^{i}$$

(row constraint)

Where ${q}_{r}^{ij}$ is the prior distribution containing the matrix of intermediate demand ${z}_{r}^{ij}$ and final demand ${f}_{r}^{i}$, which can be directly derived from the provincial SRIO table or proxy if the SRIO table is not available, as in 2015. We assume the identical technical coefficients between 2012 and 2015 and then multiply the 2015 input to get a preliminary intermediate demand. For the final demand, we first calculate the aggregated final demand by GDP minus *NE*, and then multiply the final demand distribution of 2012 as the proxy estimate. $n{e}_{r}^{i}$ represents the net export of product *i* for province *r*, which is equal to foreign export + domestic export-foreign import-domestic import by product. Foreign export and import are intermediately available from the provincial SRIO tables (for 2012 and 2017) or customs dataset (for 2015). For 2012 and 2017, we used the trade data directly from provincial SRIOTs, while the customs dataset is to estimate provincial export and import by sectors for 2015, as there are no provincial SRIO tables. ${p}_{r}^{ij}$ represents the unknown distribution dividing known prior distribution, which is the result of the GRAS; *e* is the Natural logarithm. ${X}_{r}^{j}$ represents the total input of product j for province r, while ${X}_{r}^{i}$ represents the total output of product *i* for province *r*.

### Intraregional matrix estimate

Equation 5 yields the updated provincial SRIO tables incompatibility with adjusted trade (e.g. domestic export and import) and national accounts (e.g. VA). However, the intermediate and final demands in the updated provincial SRIO table are mixed, with both the domestic and imported demands, categorised as the competitive type, while the MRIO table requires a separated matrix for domestic and imported goods. Thus, we convert the competitive table into a non-competitive table by assuming the proportion of imports in the intermediate and final demands in the SRIO table are identical^24,31^. Specifically, we introduce an indicator regional purchase coefficient (RPC) to measure the proportion of total demands supplied locally. The domestic intermediate and final demands thus can be derived by multiplying the RPC with intermediate and final demands in the updated SRIO table. Mathematically:

6 $$rp{c}_{r}^{i}=\frac{\left({x}_{r}^{i}-e{x}_{r}^{i}-s{o}_{r}^{i}\right)}{\left({x}_{r}^{i}-e{x}_{r}^{i}-s{o}_{r}^{i}+i{m}_{r}^{i}+d{o}_{r}^{i}\right)}$$

7 $${z}_{rr}^{ij}=rp{c}_{r}^{i}\times {z}_{r}^{ij}$$

8 $$f=rp{c}_{r}^{i}\times {f}_{r}^{i}$$

Similarly, we can apply the import purchase coefficient (IPC), analogous to the purchase coefficient, to derive the demands supplied from other provinces. Mathematically:

9 $$ip{c}_{r}^{i}=\frac{d{o}_{r}^{i}}{\left({x}_{r}^{i}-e{x}_{r}^{i}-s{o}_{r}^{i}+i{m}_{r}^{i}+d{o}_{r}^{i}\right)}$$

10 $$z{n}_{r}^{ij}=ip{c}_{r}^{i}\times {z}_{r}^{ij}$$

11 $$f{n}_{r}^{i}=ip{c}_{r}^{i}\times {f}_{r}^{i}$$

Where ${x}_{r}^{i}$ refers to the output of product *i* in province *r*; $e{x}_{r}^{i}$ indicates the foreign export of product *i* in province *r*; $s{o}_{r}^{i}$ refers to product *i* supply from province *r* to other provinces; $i{m}_{r}^{i}$ represents product *i* imported from other countries to province *r*; $d{o}_{r}^{i}$ represents product *i* required in province *r*. ${z}_{rr}^{ij}$ and ${f}_{rr}^{i}$ are intermediate and final demand matrix for domestic products *i* for province *r*. These matrixes are the diagonal matrix in China’s provincial MRIO table for domestic intermediate and final demands respectively. $z{n}_{r}^{ij}$ and $f{n}_{r}^{i}$ are the intermediate and final demand matrices for the imported products *i* to province *r*.

### Interregional trade matrix estimate

To obtain the trade matrix, we apply the gravity model with the observable trade data between provinces, which improves the accuracy and reliability of the interregional estimates^27,32,33^. The gravity model has been widely adopted in previous Chinese MRIO table building^17,21^. It is worth noting that the standard gravity model requires trade sample data to estimate the parameters. When the sample data are unavailable, the doubly constrained gravity model can be chosen as a reliable alternative^34^. The doubly constrained gravity model has also been used by IMPLAN to build a sub-national trade matrix for the US^35,36^. The model assumes that the trade between two regions is the function of supply and demand and the impedance in costs. Therefore, the standard gravity model is as follows:

12 $${t}_{rs}^{i}={G}^{\alpha }\frac{{\left({e}_{{\rm{ro}}}^{i}\right)}^{{\beta }_{1}}\times {\left({m}_{{\rm{os}}}^{i}\right)}^{{\beta }_{2}}}{{\left({d}_{rs}\right)}^{\gamma }}$$

Where ${t}_{rs}^{i}$ is the trade flow for commodity *i* between province *r* and province *s*; ${e}_{{\rm{ro}}}^{i}$ and ${m}_{{\rm{os}}}^{i}$ are the supply (or domestic export) from province *r* and the demand (or domestic import) of province *s*, respectively. *d*_*rs*_ is the distance between two provinces, which is the proxy for transportation costs. *β*_1_ and *β*_2_ represent the weights of the original and destination province. *γ* refers to the friction parameter. With sample trade data, the unknown coefficients for each sector (*β*_1_, *β*_2_, *γ*) can be estimated using regression. In this case, we use the railway’s interregional commodity from National Railway Statistical Data as sample data for the shippable commodity. We use the sample data as the trade flow (${t}_{rs}^{i}$) to estimate the unknown coefficients (*β*_1_, *β*_2_, *γ*) . With sample trade data, the unknown coefficients for each sector (*β*_1_, *β*_2_) can be estimated using regression. We use the sample data as the trade flow (${t}_{rs}^{i}$) to estimate unknown coefficients (*β*_1_, *β*_2_, *γ*) . As we have trade data for 11 commodities from the railway statistics, some sectors in the gravity model have to share the same coefficients. As we have trade data for 11 commodities from railway statistics, some sectors in the gravity model have to share the same coefficients (See Table S2). We show the mapping relationship in the appendix. For non-shippable commodities (e.g. service and construction), we do not set transport costs, and simply assume that they are evenly distributed based on supply and demand, as data are unavailable. For electricity transmissions, we obtained an interregional electricity transmission matrix from China Electricity Power Yearbook as electricity sample data^37^. With estimated coefficients, we can derive the initial trade matrix directly by Eq. 12. But the initial trade matrix is not in line with the constraints of row and column which are domestic export and import from the updated provincial SRIO table. We then apply the RAS model to balance the trade matrix to make it consistent with the provincial SRIO table.

Based on the balanced trade matrix, we calculate the proportion of total domestically imported products supplied from each province, defined as purchase proportion (RP), shown as:

13 $$r{p}_{rs}^{i}=\frac{{t}_{rs}^{i}}{{\sum }_{s}\,{t}_{rs}^{i}}$$

Where $r{p}_{rs}^{i}$ represents the ratio of domestic imports from province *r* to province *s* for product *i*; ${t}_{{\rm{jr}}}^{{\rm{i}}}$ refers to the trade from province *j* to province *r* for sector *i*. Therefore, the non-diagonal matrix in the MRIO table can be presented as:

14 $${z}_{rs}^{ij}=r{p}_{rs}^{i}\times z{n}_{s}^{ij}\;\;r\ne s$$

15 $${f}_{rs}^{i}=r{p}_{rs}^{i}\times f{n}_{s}^{i}\;\;r\ne s$$

So far, the diagonal matrix generates intermediate demand (${z}_{rr}^{ij}$) and final demand (${f}_{rr}^{i}$) and the non-diagonal matrix generates intermediate demand (${z}_{rs}^{ij}$) and final demand (${f}_{rs}^{i}$), which make up the provincial MRIO table.

## Data Records

**Fig. 4:**
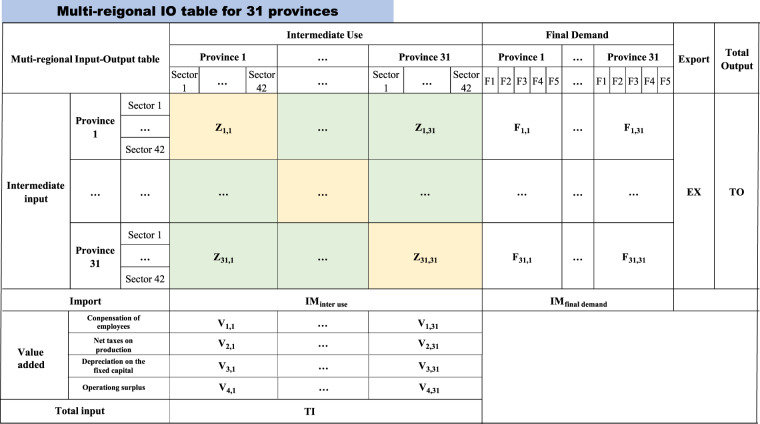
The construction of a provincial-level MRIO table.

Provincial MRIO tables illustrate the regional economic structure and interregional supply chains for 31 provinces with 42 sectors and cover China’s economic transition period for 2012, 2015 and 2017. The layout follows the standard MRIO table (Fig. 4). For each year, the MRIO table contains an intermediate matrix (1302*1302) for the 42 sectors in 31 provinces. The final demand of each province consists of 5 categories, including rural household consumption, urban household consumption, government consumption, fixed capital formation, and changes in inventories. The final demand matrix contains 1302*155 vectors for each year. In addition, foreign export contains 1302*1 vectors measuring the export for all 42 sectors in 31 provinces, while import contains 1*1302 vectors indicating the imports from other countries used by all 42 sectors in 31 provinces. Value-added includes compensation of employees, net taxes on production, depreciation of fixed capital and operating surplus, with 4*1302 vectors representing four categories of value-added for 31 provinces and 42 sectors. Notably, the national sector classification changed slightly in 2017 due to changes in the national sectoral classification. Table S1 compares sector classification for 2012, 2015 and 2017. “Other manufacturing” and “Comprehensive use of waste resources” in the 2012 & 2015 classifications are combined as “Other manufacturing and waste resources” in the 2017 classification (highlighted in bold). “Scientific research and polytechnic services” in the 2012 & 2015 classifications are separated into “scientific research” and “polytechnic services” in the 2017 classification (highlighted in bold). The MRIO tables can be freely downloaded from the China Emission Accounts and Datasets (CEADs, www.ceads.net) and Zenodo^38^. The MRIO table constructed in the paper is only at the province-level, and it can be nested into global MRIO tables for the global scale analysis (technical details seen Jiang *et al*.^39^).

## Technical Validation

### Compare with other MRIO datasets for the 2012 table

Because the most recent provincial MRIO datasets available are for 2012, we compared our MRIO table (MRIO-CEADs) for 2012 with the other two most adopted MRIO tables: the provincial MRIO table compiled by DRC (MRIO-DRC), and the provincial MRIO table compiled by CAS(MRIO-CAS). Our previously constructed table (see Table 1) is not included in this comparison, due to this table comprising only 30 sectors for 30 provinces. The format of MRIO-DRC and MRIO-CAS (42 sectors for 31 provinces) is compatible with our MRIO table (MRIO-CEADs).

Following previous work in MRIO table comparison^40^, three indicators are employed in the comparison. Specifically, we calculate the mean absolute deviation (MAD), the Isard-Romanoff similarity index (DSIM) and the absolute entropy distance (AED). These indicators measure the similarity between matrixes. MAD measures the absolute distance between each element in the two matrices; DSIM uses the relative distance instead of the absolute distance in MAD; AED is based on information theory and refers to the entropy loss between two matrices. It calculates the absolute entropy differences between two matrices. More similar to two matrices, AED is closer to zero. Here, we compare the intermediate demand matrix, representing how the sector’s production requires the other sector’s production. Mathematically:

16 $$MAD=\frac{1}{m\times n}\sum _{m}\sum _{n}\left|{z}_{ij}^{CEADs}-{z}_{ij}^{Counterpart}\right|$$

17 $$DSIM=\frac{1}{m\times n}\sum _{m}\sum _{n}\frac{\left|{z}_{ij}^{CEADs}-{z}_{ij}^{Counterpart}\right|}{\left|{z}_{ij}^{CEADs}\right|+\left|{z}_{ij}^{Counterpart}\right|}$$

18 $$AED=\left|\sum _{i}\sum _{j}{p}_{ij}ln{p}_{ij}-\sum _{i}\sum _{j}{q}_{ij}ln{q}_{ij}\right|$$

where

$${p}_{ij}=\frac{{z}_{ij}^{CEADs}}{{\sum }_{i}{\sum }_{i}{z}_{ij}^{CEADs}}$$

$${q}_{ij}=\frac{{z}_{ij}^{Counterpart}}{{\sum }_{i}{\sum }_{i}{z}_{ij}^{Counterpart}}$$

In the above, ${z}_{ij}^{CEADs}$ refers to the intermediate demand for sector *j* from sector *i* of MRIO-CEADs; ${z}_{ij}^{Counterpart}$ denotes the intermediate demand for sector *j* from sector *i* of the counterpart MRIO tables: MRIO-CAS or MRIO DRC.

Table 3 shows the results for three indicators in the comparison between the MRIO-CEADs and MRIO-DRC, and the MRIO-CEADs and MRIO-CAS. In terms of MAD, MRIO-CEADs is more similar to the MRIO CAS, with slightly less absolute distance on average (0.8 versus 0.9). Our MRIO table shows that 17 provinces are similar to them in MRIO-CAS, while 14 provinces are similar to them in MRIO-DRC. Given that MAD gives more weight to the large number, it might be more sensitive for the rich regions which have larger transactions. Our results accordingly show that indicators for rich regions such as Beijing, Shanghai, Jiangsu, Zhejiang and Guangdong are more similar to CAS. However, DSIM measuring the relative distance indicates higher similarity with MRIO-DRC where 22 provinces are closer to MRIO-DRC, despite the small gap between the two counterparts on average (27.4 with CAS versus 25.4 with DRC). AED compares the matrix in terms of information (or entropy) loss. It indicates a slight similarity with MRIO-DRC, with an average entropy loss of 13% versus 15% with CAS. Overall, three indicators might explain why our MRIO table is in the middle between two counterparts MRIO tables, while all three indicators might be similar for some provinces. For example, Chongqing in MRIO-CEADs is similar to MRIO-CAS, while Hubei in MRIO-CEADs is similar to MRIO-DRC.

**Table 3:** Comparison of three indicators, the MRIO-CEADS with the other two MRIO tables (bold value indicates smaller metric or higher similarity).

Province	MAD		DSIM		AED	
	To CAS	To DRC	To CAS	To DRC	To CAS	To DRC
Beijing	**1.42**	2.24	27.52	**26.77**	48%	**4%**
Tianjin	0.66	**0.61**	**25.26**	26.42	**3%**	4%
Hebei	1.30	**1.00**	26.77	**25.97**	16%	**7%**
Shanxi	0.48	**0.38**	28.63	**23.71**	**10%**	12%
Inner Mongolia	**0.68**	0.74	27.22	**25.59**	**8%**	12%
Liaoning	1.06	**1.04**	**25.12**	24.38	16%	**7%**
Jilin	0.45	**0.34**	29.18	**17.69**	10%	**2%**
Heilongjiang	**0.51**	0.59	27.16	**25.26**	9%	**3%**
Shanghai	**1.53**	2.13	**26.77**	27.03	30%	**2%**
Jiangsu	**2.31**	2.43	31.98	**21.44**	5%	**3%**
Zhejiang	**1.65**	1.77	26.67	**22.89**	16%	**13%**
Anhui	**1.08**	1.75	24.60	**24.38**	45%	**15%**
Fujian	0.69	**0.56**	**27.97**	29.75	**10%**	40%
Jiangxi	0.60	**0.51**	27.55	**24.98**	**4%**	15%
Shandong	2.01	**1.67**	33.47	**31.69**	**4%**	16%
Henan	**1.38**	1.46	26.01	**24.48**	19%	**13%**
Hubei	0.63	**0.46**	30.13	**28.61**	9%	**9%**
Hunan	0.77	**0.67**	27.56	**24.91**	**5%**	17%
Guangdong	**2.83**	2.93	27.37	**24.51**	**31%**	39%
Guangxi	0.40	**0.35**	26.60	**24.92**	**6%**	9%
Hainan	**0.15**	0.27	**26.86**	28.42	**4%**	33%
Chongqing	**0.46**	0.68	**26.96**	27.21	**4%**	17%
Sichuan	0.73	**0.58**	28.18	**21.06**	**2%**	6%
Guizhou	**0.25**	0.27	26.12	**24.45**	22%	**20%**
Yunnan	**0.34**	0.36	25.15	**22.32**	**6%**	13%
Tibet	**0.03**	0.04	27.72	**27.10**	21%	**17%**
Shannxi	**0.78**	0.81	25.51	**24.59**	47%	**4%**
Gansu	**0.29**	0.34	**26.13**	27.39	21%	**20%**
Qinghai	0.08	**0.06**	**27.79**	29.22	26%	**4%**
Ningxia	**0.12**	0.21	**27.68**	28.64	**4%**	12%
Xinjiang	0.34	**0.26**	27.44	**23.01**	**8%**	13%
Means	**0.8**	0.9	27.4	**25.4**	15%	**13%**
Values	17	14	9	22	15	16

Unit: 1 billion RMB for MAD; DSIM is non-unity due to being a multiplier.

**Fig. 5:**
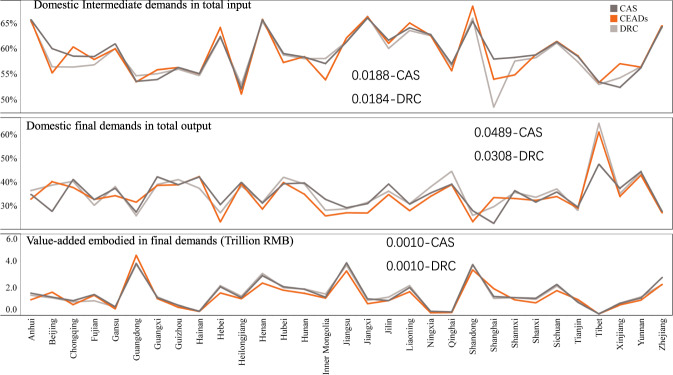
The comparison in the proportion of domestic intermediate input in the total input, proportion of the domestic final demand in the total output, and the value-added embodied in final demands. The numbers shown in the chart are the standard deviation between MRIO-CEADs with MRIO-CAS and MRIO-DRC respectively.

We then compare the province-wise proportion of domestic intermediate input to total input, the proportion of the domestic final demand to total output, and the value-added embodied in the final demand (Fig. 5). The results show that MRIO-CEADs is generally similar to the other two matrices although for some provinces differences may be more significant. In the proportion of domestic intermediate demands to total input, the biggest gap is found in Shanghai, where demand to total input is 6% less compared to the MRIO-DRC but 4% higher compared to the MRIO-CAS. The comparison in standard deviation (SD) between our MRIO table and the other two tables shows that our MRIO table is more similar to MRIO-DRC in the intermediate demand, with a tiny margin. But for domestic final demands in the total output, MRIO-CEADs is more similar to the MRIO-DRC as a general trend. The biggest gap is found in Tibet where the figure is 13% higher than in the MRIO-CAS, but only 4% higher than in the MRIO-DRC. In terms of SD, our MRIO s deviates more than in the MRIO-CAS. As for the value-added embodied in the final demands, all MRIO tables produce similar outcomes which might indicate that the main deviation occurs in the sectors with smaller value-added in the final demands, such as agriculture and mining.

### Comparison with the national SRIO table

Given that there are no MRIO tables for 2015 and 2017 compiled by other institutes, we justify our MRIO table for 2015 and 2017 by comparing it with their official national SRIO tables (Table 4). The MRIO table and SRIO table cannot be compared directly, as the national SRIO table is compiled in a competitive-import style, where imports are included in the intermediate and final demands. In contrast, the MRIO table is non-competitive style, in that intermediate and final demands only show domestically supplied demand. In the MRIO table compilation, sectoral output, value-added, export, and import in the national IO table are used in the calibration with the provincial raw data. Therefore, these national accounting data from the MRIO table are identical with the national SRIO table, and thus, the total intermediate input (input-value-added) is identical between the MRIO and SRIO tables. We then transform the competitive-import national SRIO table into a non-competitive type, assuming an identical imported ratio in both intermediate and final demands (Eq. 14). After removing the imported parts, we compare the domestic intermediate input of the national SRIO table with the input of the MRIO table. The results show that most sectors are ±5% in all three years, except for a few sectors. In 2012, Processing of petroleum, coking, processing of nuclear fuel (S11), Comprehensive use of waste resources(S23), and production and distribution of gas (S26) are outliers, being 30% higher than in the SRIO table, the most significant deviation is found in production and distribution of gas (S26 in 2015 and S25 in 2017) in 2015 and 2017. It is worth noting that these sectors in China are highly related to imports. The reason behind the uncertainty is the assumption that the import ratio is identical when transforming the competitive table into the non-competitive table. The accuracy of the ratio is, therefore, more sensitive to the sectors with higher imports. The ratio can be adjusted if more data are available to improve the model. In 2012, other datasets can also be compared with domestic input from SRIO, but the deviation is far higher. For example, Mining and washing of coal (S2) shows a deviation of 57% for MRIO-DRC and 62% for MRIO-CAS higher than in the SRIO table. The main reason for the deviation is that MRIO-DRC and MRIO-CAS are compiled based on the provincial SRIO table without being calibrated to the national one. The aggregation of provincial data are not entirely equal to the national one, as provincial data are compiled by provincial statistics agencies while national data are compiled by the national statistics bureau^41^.

**Table 4:** Comparison in domestic intermediate input between MRIO-CEADs and SRIO.

Sector	2012			2015			2017*		
	MRIO	SRIO	Gap	MRIO	SRIO	Gap	MRIO	SRIO	Gap
S1	0.356	0.350	2%	0.427	0.42	2%	0.431	0.420	3%
S2	0.109	0.107	2%	0.151	0.148	2%	0.105	0.101	4%
S3	0.045	0.044	2%	0.038	0.037	2%	0.035	0.036	−1%
S4	0.069	0.066	5%	0.094	0.092	2%	0.054	0.054	0%
S5	0.033	0.033	1%	0.059	0.058	2%	0.047	0.047	1%
S6	0.641	0.641	0%	0.849	0.846	0%	0.928	0.918	1%
S7	0.280	0.284	−1%	0.343	0.345	0%	0.299	0.297	1%
S8	0.220	0.224	−2%	0.284	0.286	−1%	0.288	0.291	−1%
S9	0.137	0.138	−1%	0.194	0.192	1%	0.192	0.191	0%
S10	0.201	0.208	−3%	0.288	0.293	−2%	0.281	0.283	−1%
S11	0.275	0.198	**39%**	0.229	0.22	4%	0.208	0.183	**13%**
S12	0.899	0.896	0%	1.206	1.207	0%	1.048	1.046	0%
S13	0.334	0.330	1%	0.494	0.49	1%	0.443	0.440	1%
S14	0.814	0.770	6%	0.879	0.867	1%	0.710	0.687	3%
S15	0.239	0.240	−1%	0.325	0.326	0%	0.307	0.305	1%
S16	0.301	0.298	1%	0.384	0.384	0%	0.312	0.314	−1%
S17	0.225	0.220	2%	0.242	0.24	1%	0.242	0.240	1%
S18	0.470	0.473	−1%	0.592	0.604	−2%	0.607	0.621	−2%
S19	0.369	0.377	−2%	0.474	0.48	−1%	0.441	0.441	0%
S20	0.369	0.392	−6%	0.487	0.525	−7%	0.575	0.617	−7%
S21	0.035	0.033	6%	0.05	0.048	5%	0.051	0.051	−1%
S22	0.018	0.019	−2%	0.026	0.026	−2%	0.036	0.036	0%
S23	0.012	0.008	**38%**	0.031	0.033	−6%	0.011	0.011	1%
S24	0.007	0.007	1%	0.009	0.009	1%	0.363	0.358	2%
S25	0.349	0.342	2%	0.444	0.442	0%	0.034	0.028	**22%**
S26	0.022	0.016	**34%**	0.043	0.038	**14%**	0.013	0.013	0%
S27	0.009	0.009	−2%	0.016	0.016	0%	1.670	1.662	0%
S28	0.970	0.972	0%	1.496	1.49	0%	0.376	0.375	0%
S29	0.208	0.213	−2%	0.371	0.376	−1%	0.531	0.533	0%
S30	0.357	0.370	−3%	0.476	0.481	−1%	0.232	0.233	0%
S31	0.132	0.133	−1%	0.17	0.17	0%	0.253	0.251	1%
S32	0.118	0.118	0%	0.181	0.176	2%	0.390	0.389	0%
S33	0.226	0.228	−1%	0.293	0.292	0%	0.195	0.195	0%
S34	0.101	0.104	−2%	0.16	0.161	−1%	0.457	0.455	0%
S35	0.209	0.214	−3%	0.403	0.404	0%	0.074	0.076	−3%
S36	0.139	0.142	−2%	0.194	0.194	0%	0.201	0.201	0%
S37	0.033	0.034	−2%	0.046	0.046	−1%	0.050	0.050	−1%
S38	0.071	0.069	2%	0.088	0.086	2%	0.123	0.119	3%
S39	0.055	0.055	0%	0.065	0.063	2%	0.102	0.100	2%
S40	0.108	0.109	−1%	0.174	0.176	−1%	0.225	0.225	0%
S41	0.033	0.033	−1%	0.045	0.045	−1%	0.063	0.062	1%
S42	0.130	0.129	1%	0.168	0.164	2%	0.213	0.208	2%

Unit: Trillion RMB.

## Supplementary information

Supplementary Information

## Data Availability

The programs used in the data generation is based on MATLAB and GAMS. The associated code can be found in Zenodo repository^38^.
