# Supplementary material for: Chinese provincial multi-regional input-output database for 2012, 2015, and 2017
Source: Sci Data. 2021 Sep 22;8:244. doi: 10.1038/s41597-021-01023-5 (PMC8458474; doi:10.1038/s41597-021-01023-5)
Supplement: Supplementary file 1 — Supplementary Information [file 41597_2021_1023_MOESM1_ESM.docx]

**Supplementary Material**

**Chinese provincial multi-regional input-output database for 2012, 2015, and 2017**

## Table S1 Sectorial classification for 2012, 2015 and 2017

| Sector | 2012 & 2015 | 2017 |
| --- | --- | --- |
| 1 | Agriculture, Forestry, Animal Husbandry, and Fishery | Agriculture, Forestry, Animal Husbandry and Fishery |
| 2 | Mining and washing of coal | Mining and washing of coal |
| 3 | Extraction of petroleum and natural gas | Extraction of petroleum and natural gas |
| 4 | Mining and processing of metal ores | Mining and processing of metal ores |
| 5 | Mining and processing of nonmetal and other ores | Mining and processing of nonmetal and other ores |
| 6 | Food and tobacco processing | Food and tobacco processing |
| 7 | Textile industry | Textile industry |
| 8 | Manufacture of leather, fur, feather and related products | Manufacture of leather, fur, feather and related products |
| 9 | Processing of timber and furniture | Processing of timber and furniture |
| 10 | Manufacture of paper, printing and articles for culture, education and sport activity | Manufacture of paper, printing and articles for culture, education and sport activity |
| 11 | Processing of petroleum, coking, processing of nuclear fuel | Processing of petroleum, coking, processing of nuclear fuel |
| 12 | Manufacture of chemical products | Manufacture of chemical products |
| 13 | Manuf. of non -metallic mineral products | Manuf. of non -metallic mineral products |
| 14 | Smelting and processing of metals | Smelting and processing of metals |
| 15 | Manufacture of metal products | Manufacture of metal products |
| 16 | Manufacture of general purpose machinery | Manufacture of general purpose machinery |
| 17 | Manufacture of special purpose machinery | Manufacture of special purpose machinery |
| 18 | Manufacture of transport equipment | Manufacture of transport equipment |
| 19 | Manufacture of electrical machinery and equipment | Manufacture of electrical machinery and equipment |
| 20 | Manufacture of communication equipment, computers and other electronic equipment | Manufacture of communication equipment, computers and other electronic equipment |
| 21 | Manufacture of measuring instruments | Manufacture of measuring instruments |
| 22 | **Other manufacturing** | **Other manufacturing and waste resources** |
| 23 | **Comprehensive use of waste resources** | Repair of metal products, machinery and equipment |
| 24 | Repair of metal products, machinery and equipment | Production and distribution of electric power and heat power |
| 25 | Production and distribution of electric power and heat power | Production and distribution of gas |
| 26 | Production and distribution of gas | Production and distribution of tap water |
| 27 | Production and distribution of tap water | Construction |
| 28 | Construction | Wholesale and retail trades |
| 29 | Wholesale and retail trades | Transport, storage, and postal services |
| 30 | Transport, storage, and postal services | Accommodation and catering |
| 31 | Accommodation and catering | Information transfer, software and information technology services |
| 32 | Information transfer, software and information technology services | Finance |
| 33 | Finance | Real estate |
| 34 | Real estate | Leasing and commercial services |
| 35 | Leasing and commercial services | **Scientific research** |
| 36 | **Scientific research and polytechnic services** | **Polytechnic services** |
| 37 | Administration of water, environment, and public facilities | Administration of water, environment, and public facilities |
| 38 | Resident, repair and other services | Resident, repair and other services |
| 39 | Education | Education |
| 40 | Health care and social work | Health care and social work |
| 41 | Culture, sports, and entertainment | Culture, sports, and entertainment |
| 42 | Public administration, social insurance, and social organizations | Public administration, social insurance, and social organizations |

## Table S2 Sector mapping in the gravity model

| **IO sector** | **Railway sample data** |
| --- | --- |
| Agriculture, Forestry, Animal Husbandry and Fishery | Food |
| Mining and washing of coal | Coal |
| Extraction of petroleum and natural gas | Petroleum |
| Mining and processing of metal ores | Metallic ore |
| Mining and processing of nonmetal and other ores | Non-metallic ore |
| Food and tobacco processing | Food |
| Textile industry | Cotton |
| Manufacture of leather, fur, feather and related products | Cotton |
| Processing of timber and furniture | Timber |
| Manufacture of paper, printing and articles for culture, education and sport activity | Timber |
| Processing of petroleum, coking, processing of nuclear fuel | Coke |
| Manufacture of chemical products | Fertiliser |
| Manuf. of non -metallic mineral products | Mineral building materials |
| Smelting and processing of metals | Steel |
| Manufacture of metal products | Steel |
| Manufacture of general purpose machinery | Steel |
| Manufacture of special purpose machinery | Steel |
| Manufacture of transport equipment | Steel |
| Manufacture of electrical machinery and equipment | Steel |
| Manufacture of communication equipment, computers and other electronic equipment | Steel |
| Manufacture of measuring instruments | Steel |
| Other manufacturing | Steel |
| Comprehensive use of waste resources | Steel |
| Repair of metal products, machinery and equipment | Steel |
| Production and distribution of electric power and heat power | Electricity |
